# Polymorphism and Perfection in Crystallization of Hard Sphere Polymers

**DOI:** 10.3390/polym14204435

**Published:** 2022-10-20

**Authors:** Miguel Herranz, Katerina Foteinopoulou, Nikos Ch. Karayiannis, Manuel Laso

**Affiliations:** Institute for Optoelectronic Systems and Microtechnology (ISOM) and Escuela Técnica Superior de Ingenieros Industriales (ETSII), Universidad Politécnica de Madrid (UPM), José Gutierrez Abascal 2, 28006 Madrid, Spain

**Keywords:** polymorphism, perfection, crystallization, hard sphere, Monte Carlo, hexagonal close packed, face centered cubic, fivefold, phase transition, random walk

## Abstract

We present results on polymorphism and perfection, as observed in the spontaneous crystallization of freely jointed polymers of hard spheres, obtained in an unprecedentedly long Monte Carlo (MC) simulation on a system of 54 chains of 1000 monomers. Starting from a purely amorphous configuration, after an initial dominance of the hexagonal closed packed (HCP) polymorph and a transitory random hexagonal close packed (rHCP) morphology, the system crystallizes in a final, stable, face centered cubic (FCC) crystal of very high perfection. An analysis of chain conformational characteristics, of the spatial distribution of monomers and of the volume accessible to them shows that the phase transition is caused by an increase in translational entropy that is larger than the loss of conformational entropy of the chains in the crystal, compared to the amorphous state. In spite of the significant local re-arrangements, as reflected in the bending and torsion angle distributions, the average chain size remains unaltered during crystallization. Polymers in the crystal adopt ideal random walk statistics as their great length renders local conformational details, imposed by the geometry of the FCC crystal, irrelevant.

## 1. Introduction

The importance of crystallization in physics, materials, life science, and technology cannot be sufficiently emphasized. It plays a major role in the pharmaceutical industry [1], the storage of clean fuels such as hydrogen [2], and catalytic processes [3], among other industrial processes [4]. Furthermore, ordered structures of polymer-based physical and chemical systems are of paramount importance in the development of novel solar cells [5], semiconductors [6], biological materials [7], or conventional plastics [8]. Due to its high complexity, crystallization remains a topic surrounded frequently by scientific controversy and debate, especially since the classical view is considered to be too simple to cover a wide range of physical systems and conditions [9,10,11]. The basics of crystallization have been studied, among other systems, on simple spherical hard colloids, due to their suitability as macroscopic models, as they can be easily probed, and they bear properties and characteristics that can be tailored within wide ranges [12,13].

Extensive and careful experimental work has unmistakably demonstrated the difficulty of obtaining quite perfect crystals [14,15,16]. The identification of competing ordered structures of colloidal crystals has been carried out using methods such as fluorescence confocal scanning laser microscopy [17], small-angle synchrotron X-ray diffraction [18], laser scanning confocal microscopy [15], or light scattering [19]. In many of these experimental studies, the random hexagonal close packed (rHCP) structure prevails as the final ordered structure. It has also been shown that gravity and microgravity play a significant role in the crystallization of colloids [14,20,21,22,23]. In many cases, the aging of the structures of colloidal spheres leads to a slow transition from the rHCP morphology to the theoretically expected, and thermodynamically more stable, face centered cubic (FCC) structure of varying degrees of perfection [24,25,26].

The existing experimental efforts have been accompanied by numerous analytical and simulation works [27,28,29,30,31,32,33]. The experimentally observed sluggishness of the rHCP→FCC transformation also appears in simulations [34]. Obtaining a stable crystal of well defined, close packed character is still a very challenging task, even for the simplest possible system realization, that of monomeric hard spheres (HS). This competition of the close packed HCP and FCC crystals has been studied from the perspectives of nucleation [35,36,37], entropy (free energy) difference [34,38,39,40,41,42], geometric arguments [43], and Ostwald’s rule of steps [44], among others. Theoretical works have been accompanied by simulations, as in [42,43,45,46,47,48].

The available estimates of the entropy (directly proportional to free energy for hard spheres) demonstate an advantage of FCC against HCP that ranges between $9\times 10^{-4}$ and $50\times 10^{-4}$ per particle (expressed in terms of Boltzmann’s constant, *k*) [38,40,41,42,46]. The variation is attributable primarily to the methods used, and to a lesser extent, to the conditions under which it is calculated: ΔS seems to vary by about 25–30% between the melting transition and the maximum density. It is the smallness of this value that is responsible for the difficulty of obtaining neat crystals of the stable FCC polymorph, both experimentally and computationally. As a consequence, it is not surprising that the vast majority of isochoric simulations, starting from predominately amorphous monomeric HS packings, result in highly defective ordered structures of rHCP character [28,30,49,50]. The body of simulation works where FCC-like crystals are obtained is very limited [51,52]. The investigation of the FCC–HCP competition has been extended in simulations to the study of the effect of gravity [53], crystallization from seeds [36], sedimentation [54], template-assisted crystallization [55,56,57], or crystallization on surfaces and interfaces [58,59,60] .

Research studies addressing the crystallization of hard spheres forming linear sequences of chains are very sparse compared to the monomeric (single) HS analogs. Experiments on linear polymers of hard spheres are very challenging. In spite of the granular, colloidal, or droplet polymers being significantly less well explored than the “traditional” ones, over the years, there have been significant advances in their synthesis and characterization [61,62,63,64,65]. From the perspective of theory and simulation [66,67], emphasis is placed on the phase transition of semi-flexible [68,69,70] or flexible [71,72] chains of hard spheres, while recently, it was demonstrated that the use of block copolymers leads to HCP stable phases [73,74,75]. The stabilization of HCP colloidal structures has been investigated through the insertion of polymers [76,77]. In spite of these advances, crystal perfection and the relative stability of crystals of long, entangled HS polymers are still considered to be uncharted territory.

The very long relaxation times are responsible for the difficulty of preparing crystals of polymers of reasonable quality in the laboratory. It is no exaggeration to say that high-quality bulk polymer crystals with a well defined habit remain a laboratory curiosity, although a very valuable one, for they provide fundamental information on the unique characteristics of polymer crystallization. Most crystalline and semicrystalline polymers consist of huge assemblies of imperfect crystals, often and unavoidably combined with amorphous regions. Focusing on the computational work, the large size and concomitant sluggish dynamics of polymers present researchers with unusual challenges. Major conformational rearrangements involving the slowest modes, which play a key role in the formation of polymer crystals, can be well beyond the reach of deterministic methods.

Monte Carlo (MC) methods do not suffer from the slow dynamics associated with large molecular size, as in Molecular Dynamics (MD). The most advanced MC methods are precisely based on highly non-physical moves that allow for rapid equilibration and robust sampling in the configuration space [78,79]. This advantage is obviously offset by the loss of dynamic information, although methods such as Kinetic Monte Carlo (KMC) [80,81] do offer a reasonable compromise when the rates of individual events are known in advance. Developing MC methods that correctly and efficiently sample polymer conformational space is not a trivial matter, especially for very long chains at very high concentrations. In spite of these difficulties, a wide variety of increasingly more efficient MC methods have been developed over the last few decades [78,79,82,83,84,85,86,87,88]. The work to be reported in this manuscript is based on a powerful suite of advanced MC moves [89], which in the past has enabled us to observe the entropy-driven athermal polymer crystallization for the first time [71], and to identify and analyze the factors that affect the phenomenon, including chain length and its distribution [71,90], the presence of bond gaps or tangency [91], and confinement in one [92] or all three [93] dimensions.

In the present contribution, unprecedentedly long Monte Carlo simulations allow for the study of polymorphism and perfection in very long chains of hard spheres. These isochoric simulations start from an isotropic amorphous packing, and after a transient dominance of the HCP polymorph and the successive establishment of rHCP morphologies of various levels of ordering, they eventually reach a final FCC crystalline state of remarkable perfection. We gauge the established crystal structures and the fivefold local symmetry, and analyze the bond geometry and global sizes of the hard-sphere chains in each region of the phase transition. The entropic origins of crystallization, strongly related to the structural rearrangements of the local environment around each site, are also described and quantified.

In a companion paper [94] we support the computationally observed stability of the FCC polymorph by means of quantitative analytic calculations.

## 2. Methodology

We adopt the freely jointed model of linear chains comprising hard sphere monomers with uniform diameter σ, which is taken as the unit length. The pair-wise energy, $u_{HS}\left(r_{ij}\right)$, is given by the equation:

(1) $$u_{HS}\left(r_{ij}\right)=\left\{\begin{array}{c} 0,r_{ij}\geq \sigma \\ \infty ,r_{ij}<\sigma \; \end{array}\right.$$

where $r_{ij}$ is the distance between the centers of monomers *i* and *j*. This is the only type of interaction; neither bending nor torsional potentials are applied to successive monomers along the chain backbone. For numerical convenience, bond lengths, $b_{len}$, are allowed to vary uniformly in the interval $b_{len}\in \left[\sigma ,\sigma +db\right]$, where db is the maximum bond tolerance (gap) between two successive monomers, and it is set equal to 0.13σ. $\langle b_{len}\rangle$ corresponds to the average bond length, where 〈〉 denote the average over all bonds for a given set of system configurations (frames).

The simulations are conducted using the *Simu-D* simulator-descriptor suite [89] in the isochoric, semigrand ensemble $\left[VTN_{sites}\mu^{*}\right]$. In the $\left[VTN_{sites}\mu^{*}\right]$ ensemble [95] underlying the present calculation, chain lengths obey a given distribution, which is enforced via means of the chemical potentials $\mu^{*}$, and are allowed to fluctuate within a predetermined range. The practical implementation for the uniform and Flory chain length distributions is explained in detail in the Appendix of Ref. [96]. For athermal systems, the formal conjugate pair of the variables T,U is inactive (see [95] for details). The system under study comprises N=54 chains of average size $l_{av}=1000$, leading to a total of $N_{sites}=54,000$ monomers or sites. Here, a flat (uniform) chain length distribution is chosen within the interval $\left[l_{min},l_{max}\right]$ with $l_{min}=600$ and $l_{max}=1400$, as a requirement for the application of specific algorithms (see below). As will be shown in the results section, this chain length range lies deep in the polymeric regime. The uniform distribution is selected over the Flory one, as it allows for the robust sampling of the long-range chain characteristics in the whole interval $\left[l_{min},l_{max}\right]$.

The core of the Monte Carlo suite consists of chain-connectivity-altering moves (CCAMs) that allow for the robust equilibration of the system, even at very high volume fractions [96,97,98], up to the maximally random jammed state [99]. CCAMs are accompanied by more standard MC moves. The following attempt probabilities for each move have been used: (i) rotation (10%), (ii) reptation (10%), (iii) flip (34.8%), (iv) intermolecular reptation (25%), (v) configurational bias (20%), (vi) simplified end-bridging, sEB (0.1%), and (vii) simplified intermolecular end-bridging, sIEB (0.1%). Neither cluster [100] nor identity exchange moves [89] are incorporated here.

The initial configuration is generated through the progressive shrinkage of a very dilute configuration that meets the constraints imposed by chain connectivity until a desired packing density (volume fraction) of φ=0.56 is reached [89], or $\varphi^{*}=\frac{\varphi }{\varphi^{FCC}}=0.756$ relative to the maximum compacity of the FCC or HCP crystals ($\varphi^{FCC}=\varphi^{HCP}=0.7405$). The cubic simulation cell is subjected to periodic boundary conditions in all directions. Due to the high density, a configurational MC bias pattern is used throughout, with $n_{dis}=50$ candidate configurations being attempted per local move, as explained in detail in [89,99]. The isochoric simulation is carried out over $1.4\times 10^{12}$ MC steps, and system configurations are recorded every $10^{8}$ MC steps. Due to the very large system size, the time required to establish crystal perfection was close to 4 years of continuous wall-clock CPU time on a single Intel i5 processor with 32 Gb of memory.

The core post-processing tool of the work is the Characteristic Crystallographic Element (CCE norm), as implemented in the descriptor part of the *Simu-D* software [89]. This descriptor of structural order gauges both the radial and orientational similarity of the local environment of a monomer with respect to the reference crystals in two or three dimensions [101,102]. To this end, a tessellation into Voronoi cells (VC) around all monomers of the system is carried out (by means of the voro++ software [103]), and as a first step, the closest neighbors are identified for each monomer. The CCE norm is then computed for each monomer by carrying out the geometric symmetry operations of the point group of several candidate reference crystals (e.g., FCC and HCP) and evaluating the deviation from geometric invariance. The CCE norm, $\varepsilon_{j}^{X}$ is assigned to each monomer, *j*, which is a measure of the deviation of its environment with respect to each reference crystal, *X*. The closer the value of the norm to zero, the greater the similarity of the monomer’s environment to the reference crystal. A type is assigned to each monomer, depending on its structural similarity to a given characteristic point group (crystalline or not), by checking its CCE-norm against a threshold value, set here at $\varepsilon^{thres}=0.3$. This value was found to ensure selective discrimination among competing polymorphs for the system at hand. We calculate this CCE norm for each monomer at each MC frame, independently of which chain they belong to, in order to quantitatively analyze the evolution of crystallinity and the competition among polymorphs [101,102].

System configurations are tested against all reference 3D crystals implemented in *Simu-D*: hexagonal close packed (HCP), face centered cubic (FCC), body centered cubic (BCC), simple hexagonal (HEX), but also with respect to the non-crystallographic fivefold (FIV) local symmetry. The salient differences in the crystallographic operations and point groups, as well as in the shape and size of the corresponding Voronoi polyhedra for each reference crystal, are analyzed in detail in Ref. [102]. Only four different types of monomers are detected along the entire simulation:

- Monomers with first neighbors whose arrangement conforms to the point symmetry group m3m of the face centered cubic crystal (FCC sites),
- Monomers with first neighbors whose positions conform to the point group $\bar{6}m2$ of the hexagonal close packed crystal (HCP sites),
- Monomers whose first neighbors conform to the point group 5 of a non-crystallographic fivefold axis (FIV sites),
- Monomers with first neighbors that are not arranged according to any kind of symmetry (apart from the identity *E*), either crystallographic or non-crystallographic (amorphous, denoted AMO).

In all of the analyzed sites and over all of the recorded configurations (frames), no instances of sites with BCC or HEX symmetry are found. Based on the CCE-norm, an order parameter, $S^{X}\in \left[0,1\right]$ for each reference point group type X∈ [HCP, FCC, FIV] is defined as:

$$\begin{array}{c} S^{X}=\int_{0}^{\varepsilon^{thres}}P\left(\varepsilon^{X}\right)\;d\varepsilon^{X}\; \end{array}$$

where $P(\varepsilon^{X})$ is the probability distribution function for the CCE norm of the *X* reference point group symmetry. In addition, given that only FCC and HCP crystalline sites exist in the system configurations $S^{AMO}=1-S^{HCP}-S^{FCC}-S^{FIV}$. As neither AMO nor FIV sites contribute, the total crystallinity is given simply by:

$$\begin{array}{c} \tau_{c}=S^{FCC}+S^{HCP}\; \end{array}$$

## 3. Results

As stated earlier, the simulation reaches $1.4\times 10^{12}$ MC steps, with one configuration for every $10^{8}$ MC steps being stored, for a total of 14,000 configurations (or *frames* or *snapshots*). This separation between successive frames was found to be sufficient to ensure frame decorrelation. The number of $10^{8}$ MC steps between successive frames corresponds to an average of $1.85\times 10^{6}$ MC moves per chain between successive frames.

### 3.1. Evolution of Crystallinity

**Figure 1:**
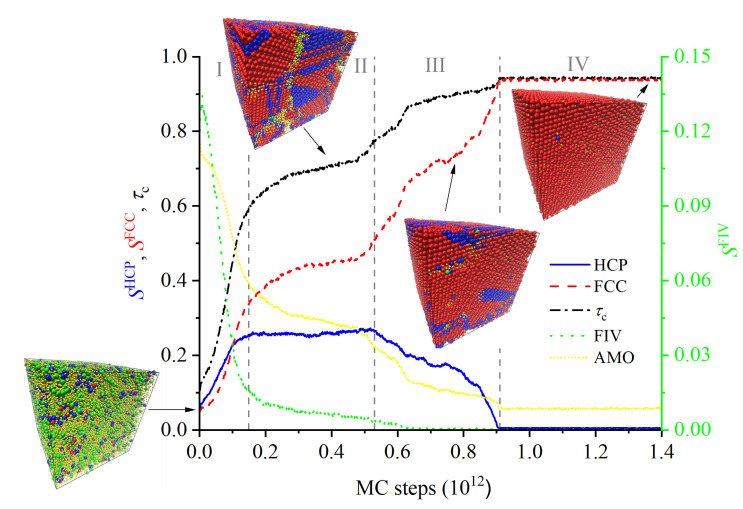
Evolution of the number fractions, $S^{X},X\in \left[\mathrm{HCP},\;\mathrm{FCC},\;\mathrm{FIV},\;\mathrm{AMO}\right]$ of sites of each type along the MC calculation. Fivefold-order parameter is shown on the right *y*-axis. Roman numerals and dashed vertical lines denote the four distinct regions in the evolution of the morphology (see main text). Also shown are perspective visualizations of representative frames in the four regimes. Spherical monomers are colored following the same convention as the curves according to their point group symmetry/crystal type: HCP: blue, FCC: red , FIV: green, and AMO: yellow. The corresponding curves are represented by solid (HCP), dashed (FCC), dotted (FIV), dashed-dotted ($\tau_{c}$), and short-dotted (AMO) styles. The correspondences of the color scheme and line style to the HCP/FCC/FIV structures of this figure are valid for the rest of the manuscript. For the system snapshots, the corresponding order parameters are: Frame 1 (Region I): $S^{HCP}=0.0548$, $S^{FCC}=0.0406$, $S^{FIV}=0.108$; Frame 4000 (Region II): $S^{HCP}=0.259$, $S^{FCC}=0.452$, $S^{FIV}=0.006$; Frame 8000 (Region III): $S^{HCP}=0.149$, $S^{FCC}=0.751$, $S^{FIV}=5\times 10^{-4}$; Frame 14,000 (Region IV): $S^{HCP}=0.005$, $S^{FCC}=0.940$, $S^{FIV}=9\times 10^{-5}$.

**Figure 2:**
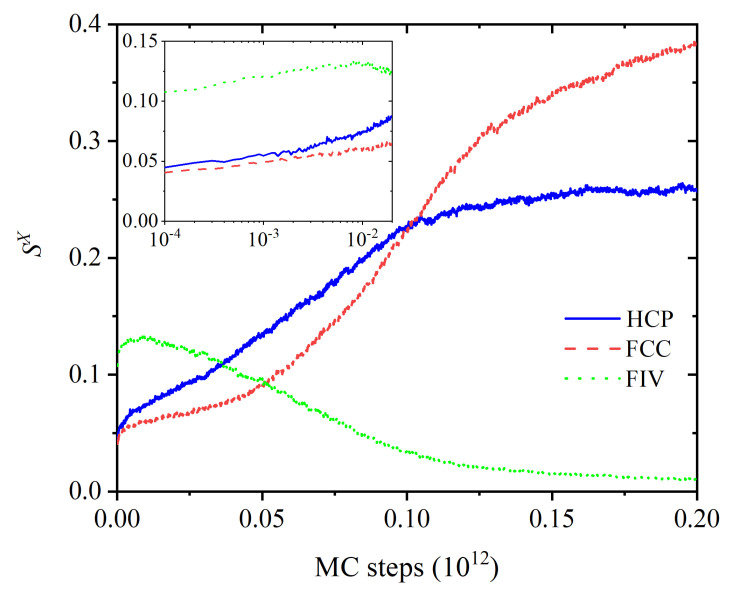
Early evolution of site fractions $S^{X},X\in \left[\mathrm{HCP},\;\mathrm{FCC},\;\mathrm{FIV}\right]$ in the first 2000 frames ($2\times 10^{11}$ MC steps). Inset: zoom on the first 200 frames ($2\times 10^{10}$ MC steps) in log-linear scale.

Starting from a purely amorphous, statistically homogeneous configuration, the system evolves through intermediate states until a stable polymorph of remarkable perfection is formed. The evolution of the individual order parameters and total crystalinity as a function of the MC steps is shown in Figure 1 and Figure 2. The intermediate states, which appear before the MC calculation settles in an equilibrated FCC polymorph, are characterized by a spatial distribution of regions of varied crystallographic nature, which evolve both in size and perfection along the simulation. Figure 1 covers the whole trajectory while Figure 2 focuses on the early part. The fraction of amorphous sites, $S^{\mathrm{AMO}}$, quantifies the degree of disorder in the system, to be contrasted against the degree of crystallinity, $\tau_{c}$. The system evolution can be conveniently split in four qualitatively different regions (Figure 1), numbered I through IV. Region I: MC steps $1\times 10^{8}$–$1.5\times 10^{11}$ (or equivalently frames 1–1500), II: MC steps $1.5\times 10^{11}$–$5.3\times 10^{11}$ (frames 1500–5300), III: $5.3\times 10^{11}$–$9.1\times 10^{11}$ (frames 5300–9100), IV: $9.1\times 10^{11}$–$1.4\times 10^{12}$ (frames 9100–14,000). From now on, except if otherwise stated, the frame numbers are quoted as rounded to the nearest 100, and are understood to be approximate, just as the boundaries between regions are not strictly sharp. Throughout the manuscript we will use kinetic terms such as “rate”, “fast”, etc., for brevity and simplicity, in order to describe the evolution of the system measured in terms of MC steps, but without claiming any truly kinetic or dynamic meaning.

In Region I, starting from the initial amorphous configuration, a rapidly growing number of monomers spontaneously develop crystalline characters, as evidenced by the steep drop in the AMO curve and the simultaneous growth of the number of FCC and HCP sites. As a matter of fact, 8.4% of all sites already have definite HCP or FCC character, and 11% show FIV character in the very first frame ($10^{8}$ MC steps). This is the reason for the curves in Figure 1 and Figure 2 apparently not starting at 0 (HCP, FCC, and FIV) or 1 (AMO), and is a consequence of the great length of the calculation: alone in the first frame, $1.85\times 10^{3}$ MC moves have been carried out, on average, for each of the $N_{sites}=54,000$ monomers. These observations are also qualitatively consistent with past simulations of dense random packings of monomers [49,50] and polymers [91,104,105], where the population of fivefold sites can exceed the sum of sites with HCP or FCC character.

**Figure 3:**
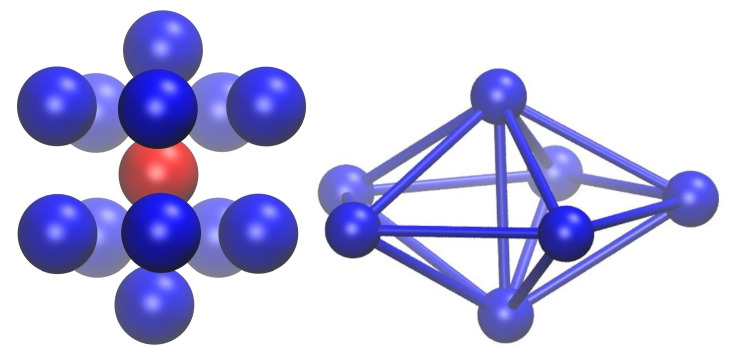
(**Left panel**) First neighbor shell for a representative site of FIV character ($\varepsilon_{14,717}^{FIV}=0.05$) in frame 1276 (Region I) of the MC simulation. The 12 neighbor atoms are marked in blue, and the reference atom in red. Ten four-monomer tetrahedra share a common fivefold axis and the reference site (marked in red).The non-crystallographic fivefold axis runs through the topmost, the middle (red), and the bottom-most sites, i.e., vertically on the page. (**Right panel**) The corresponding pentagonal bipyramid. Image created with the VMD visualization software [111].

After growing to approximately ≈10% within the first frame, the noncrystallographic FIV population remains largest in the first few frames, and then it drops steadily in parallel with the population of AMO sites, in favor of the FCC and HCP sites. The initial nucleation sites are transformed into compact assemblies of four sites of very approximate tetrahedral shape. The appearance of FIV sites is favored by the ease with which these four-monomer tetrahedra can be arranged in a variety of almost compact clusters that fill space efficiently at a small scale, like the pentagonal bipyramid shown on the right panel of Figure 3. The reason for the metastability of such arrangements is primarily geometric: at the smallest scale, five four-site tetrahedra can be arranged around a common axis so that they share an edge and build a seven-monomer bipyramid of almost, but not quite, exact fivefold symmetry, the fivefold axis being the common edge. By the ordered accretion of further elemental tetrahedra, structures of pentagonal symmetry and varying complexity [106,107,108,109] appear as metastable, long-lived morphologies in simulations. The left panel of Figure 3 shows the first neighbor shell of a representative site of FIV character ($\varepsilon_{14717}^{FIV}=0.05$), as is spontaneously formed in Region I. Two of the pentagonal bipyramids described above share one site (marked in red in Figure 3) and also have a common, single fivefold axis, running vertically on the paper in this figure. This 13-site structure and other similar arrangements [110] are favored with respect to local disorder, because they lead to a localized increase in available volume and thus in translational entropy.

**Figure 4:**
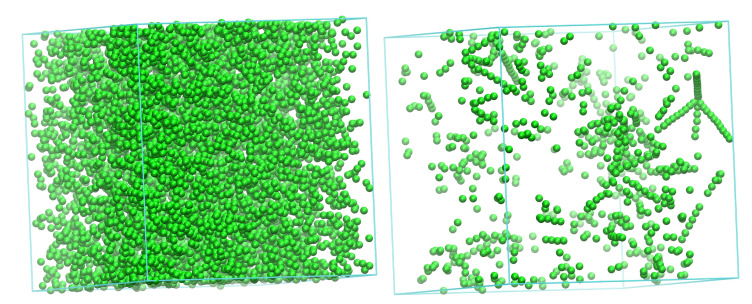
Spatial distribution of FIV sites in (**Left**): Region I (frame 125, $S^{FIV}=0.129$) and (**Right**): Region II (frame 1622, $S^{FIV}=0.014$). Image created with the VMD visualization software [111].

Initially, FIV sites appear abundantly and are randomly distributed in the simulation cell, but as their population subsequently decreases, they develop a remarkably non-homogeneous distribution in space. The left panel of Figure 4 shows this spatial distribution of sites with FIV symmetry at a configuration within Region I (frame 125). At this stage, most of the numerous FIV sites ($S^{FIV}=0.129$) are still isolated, although the first traces of the linear assemblies of FIV sites are already visible. At a later stage of the simulation (frame 1622, early in Region II), as can be seen in the right panel of Figure 4, the FIV population is significantly lower ($S^{FIV}=0.014$) and the majority is organized in specific geometric patterns that are entirely analogous to those observed in the past [108,109].

**Figure 5:**
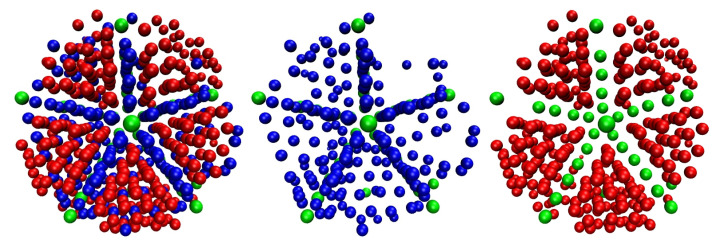
Typical cyclic twin structure (**left panel**) at frame 1622, early in Region II. View is along the twin axis [110] (perpendicular to the page). The twin axis is occupied by sites with fivefold symmetry; sectors are of mixed HCP (**middle panel**) and FCC (**right panel**) characters. Only a spherical portion of the simulation cell around the fivefold axis is depicted. Radius of spheres is reduced for clarity. Image created with the VMD visualization software [111].

Pentatwin formation is especially favorable due to the similarity between the pentagonal 72${}^{\circ }$ angle and the value of the dihedral angle 70.53${}^{\circ }$ between two faces of the regular tetrahedron. A gap of $360^{\circ }-5\times 70.53^{\circ }=7.35^{\circ }$ is left when five equally sized tetrahedra share an edge. Such tetrahedral nuclei appear easily via site accretion around the initial, minimal, four-monomer tetrahedra of Region I, and five such clusters form an almost perfect pentagonal dipyramid. Interestingly, the small angular mismatch of 7.35${}^{\circ }$ is not taken up by non-crystalline monomers as five equal, wedge-like gaps $\approx \frac{1}{5}7.35^{\circ }$ placed among the five twins, but the whole mismatch remains confined between two adjacent tetrahedra that are less perfect than the remaining three (see the rightmost two sectors in the middle panel of Figure 5). Because of the impossibility of compactly tesselating the 3D space using regular tetrahedra [112], noncrystallographic FIV sites are a transient phenomenon and tend to disappear in favor of compact structures of crystallographic symmetry. The simulation does indeed evolve the system into configurations of higher crystallographic FCC or HCP character. Consequently, a marked decrease in the number of FIV sites is observed in Region I, while total crystallinity increases rapidly. A video showing the FIV sites’ evolution in Regions I and II (first 5000 frames) can be found in the Supplementary Material.

The growth rates of both FCC and HCP crystalline sites are roughly similar in Region I, as shown in both the main and inset panels of Figure 2. The main reason is the high geometric similarity in the structures of HCP and FCC: both can be obtained via the stacking of two-dimensional, hexagonally close packed layers, differing only in the stacking sequence, …A-B-A-B-… for HCP and …A-B-C-A-B-C-… for FCC. In the case of monomeric hard spheres, these two polymorphs (and all rHCP variations on the stacking theme) are separated by a very small entropic difference [34,38,39,40,41,42], i.e., there is no initial overwhelming preference for the formation of the one over the other. As a consequence, and as long as a sufficient pool of amorphous sites is available, both polymorphs have roughly similar probabilities of being formed. The very large size of the simulation cell offers abundant volume for the independent growth of the nuclei of both types of crystals, i.e., there is plenty of available space for both the FCC and HCP crystalline regions to grow, as described above, without direct mutual competition.

**Figure 6:**
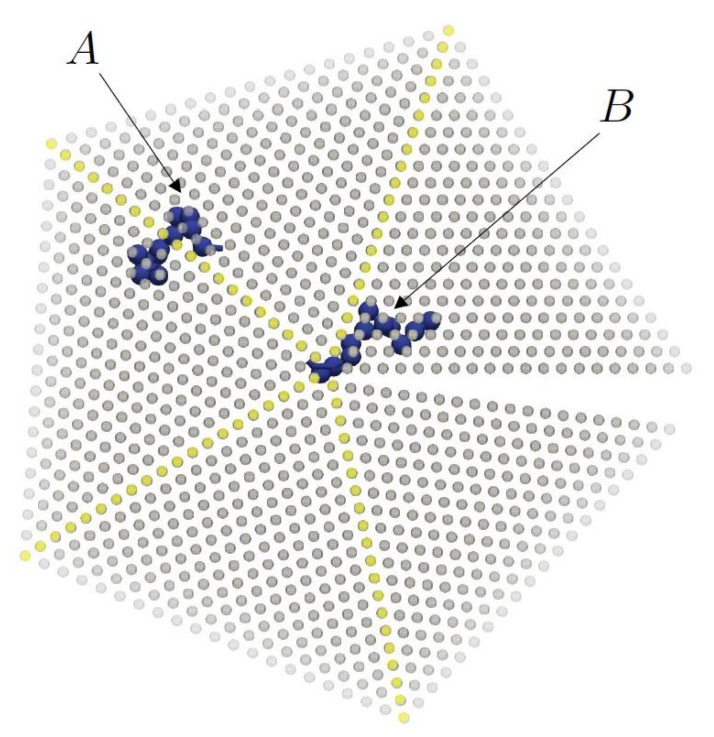
In a cyclic pentatwin (pentagonal bipyramid), chains that span the boundary between twin sectors (**A**) or that pass through the twin axis (**B**) lose configurational entropy with respect to those in a bulk, untwinned crystal, as explained in detail in [113]. The white (empty) horizontal wedge is the angular gap of $7.35^{\circ }$ left when five equally sized tetrahedra are arranged cyclically around the twin axis (perpendicular to the paper in this figure).

In Region II, the rapid decrease in the number of AMO and FIV, and the acompanying growth in the crystalline FCC and HCP sites slows down appreciably, especially when compared to the trends observed in Region I. In this region, the numbers of FCC, FIV, and especially HCP sites remain much more constant, while the total crystallinity already exceeds ≈60%. Region II is characterized by the competition between the HCP and FCC crystalline regions. Region II can be seen as an induction period, and is, in a sense, a crucial phase and a stringent test of the efficiency of the MC scheme to properly and efficiently evolve the system towards the stable FCC polymorph, which is separated from HCP and the various rHCP structures by tiny free energy differences. The small fraction ($O(10^{-2})$) of FIV sites remaining in Region II have a different character from those in Region I, and they are not homogeneously distributed in space any more. After the rapid disappearance of the initial isolated FIV sites of Region I, the remaining FIV sites associate in almost perfectly linear clusters of ≈O(10) sites. These linear assemblies of FIV sites are the axes of cyclically twinned crystalline domains (see the schematic in Figure 6). In the ideal case, the pentatwins consist of five tetrahedral sectors, each made up of between a few dozens and a hundred monomers. Five such tetrahedra assemble in cyclic twin structures, the axis of which consists of sites with FIV symmetry, while the sectors can have mixed FCC and/or HCP character.

In Region II, the number of HCP sites reaches a plateau, while the number of FCC monomers grows slowly and preferentially at the expense of both AMO and FIV sites. The total crystallinity in Region II is already appreciable, and most HCP and FCC clusters are contiguous, so that a conversion of crystalline HCP sites into FCC ones would be geometrically possible. In spite of this, it turns out to be easier to generate new FCC sites from the pool of available AMO sites than to convert neighboring HCP sites into FCC ones. This is consistent with the entropy difference between the amorphous state and both FCC and HCP crystals being larger than the entropy difference between FCC and HCP [94].

The difficulty of the FCC↔HCP interconversion is also a consequence of the appreciable metastability of the pentatwins: so long as they exist, the composition (HCP and FCC) of their sectors remains basically unchanged, so that HCP sites residing in pentatwin sectors are as immune to transformation into FCC as the twin itself [72]. This is the reason for why the proportion of HCP sites does not vary appreciably, as long as pentatwins exist.

There is also an extra factor, absent in monomeric systems and specific to chain molecules, that renders most mechanisms of FCC↔HCP interconversion for chains more difficult than for monomeric systems. At first sight, the most straightforward mechanism for HCP→FCC conversion is the rearrangement of the stacking … A-B-A-B-A-B-…→… A-B-C-A-B-C-…, which would allow FCC crystalline domains to grow at the expense of contiguous HCP domains. This rearrangement is, however, totally suppressed in polymer systems, because it entails a lateral slide of the (0001) HCP planes along the 〈12·0〉 directions. This slide, which is energetically expensive but allowed in monomeric systems [39], is not possible, even in principle in a polymer system, without breaking the chain backbones.

Easy pentatwin formation, metastability and slide suppression are the main reasons for the relative long life of the HCP polymorph in Region II. In spite of the efficiency of the MC scheme, it takes a considerable number of steps in the induction regime (plateau Region II in Figure 1), which correspond to months in wall-clock time, to initiate the final HCP→FCC conversion. The axes of these pentatwins appear as linear assemblies of sites of FIV symmetry, such as those in the right panel of Figure 4, which show the spatial distribution of sites with FIV symmetry at frame 1622 in Region II. These compact, non-crystallographic aggregates of tetrahedra also show up conspicuously as long-lived metastable states, even for monomeric systems. They represent intermediate states of appreciable stability. Their stability stems from the fact that the tetrahedra that compose the pentatwin can all grow simultaneously and independently of each other by accreting further sites from the periphery of the pentatwin. For every new surface layer accreted on the pentatwin, its axis, i.e., the linear assembly of FIV sites, grows by one site, and the number of sites in the pentatwin by $5n_{FIV}\left(n_{FIV}-1\right)+1$ sites, where $n_{FIV}$ is the number of FIV sites along the twin axis, without necessitating any change in the existing structure. As a consequence of their easy formation and growth, ordered, noncrystallographic, metastable aggregates of FIV sites are found not to disappear, even in very long MD simulations of monomeric or polymeric packings [49,50,104,105].

The length of the induction period (Region II) is primarily determined by the slow disappearance of metastable structures having linear assemblies of FIV sites as their axes. The disappearance of metastable pentatwins is dictated by the impossibility to indefinitely grow into a periodic crystalline structure. For completeness’ sake: the exotic possibility of the appearance of quasicrystalline structures has never been observed in our simulations of HS packings. In the present work, the difficulty of evolving the system out of this metastable situation, i.e., of spontaneously resolving the HCP–FCC competition, is marked by the slowdown in the evolution of all types of sites, i.e., the relative flatness of all curves in Region II, whose duration, measured in wallclock time, was about two years. The disappearance of FIV sites signals the end of Region II. It is only when the last linear FIV assemblies disappear that crystals resume their growth. The HCP–FCC competition is by then resolved, and the HCP regions start transforming into the stable FCC polymorph.

The mechanism of the disappearance of linear FIV assemblies in crystals of polymers is not just due to fluctuations brought about by the localized rearrangements of sites, as is the case in systems of monomeric hard spheres. In the case of chain molecules, the cyclic pentatwin incurs an extra entropic penalty with respect to the untwinned HCP or FCC crystals, because its sites are not individual spheres, but monomers of a polymeric chain. There exists an entropic penalty for chains in a cyclic pentatwin caused by the reduced number of available chain conformations with respect to those in a untwinned crystal. In the pentatwin, some chains are forced to span the (111) boundaries between sectors (marked with “A” in Figure 6), or to occupy sites in the [110] twinning axis (the linear FIV assembly, perpendicular to the plane of the paper marked with “B” in Figure 6). These two geometric situations force some of the torsional angles along the chain to adopt highly improbable values, which entails a decrease in entropy.

In [113], we evaluated the entropic difference between a twinned and a crystalline, but untwinned (bulk) system of hard-sphere chains, and found it to quantitatively explain the almost complete absence of FIV sites in properly equilibrated crystals of hard-sphere polymeric chains. The magnitudes of these entropic penalties in a pentatwin have been estimated to be −0.002k per monomer for chains spanning (111) sector boundaries, and −0.074k per monomer for chains that pass through the [110] twin axis. The former value is comparable to the entropy difference between the HCP and FCC polymorphs for equal monomeric spheres [34,38,39,40,41,42], while the latter is ≈35 times larger. These two entropic penalties are thus sufficient to destabilize cyclic twins for chains, in agreement with previous simulation results on hard-sphere chains [71,90,91,114].

Once the induction phase is completed, the numbers of FIV and HCP sites in Region III both drop to zero, while the number of FCC monomers grows monotonically, up to a maximum of ≈94%. At the end of Region III, the populations of all site types reach an equilibrium state, which signals the onset of the production phase (Region IV), in which all of the properties fluctuate about well defined average values. Each of the frames in Region IV is a microstate of the macroscopic, crystalline, stable polymorph, generated with the correct probability from the $\left[VTN_{sites}\mu^{*}\right]$ ensemble. The difference in entropy (and thus, the stability) between the stable polymorph and all other macrostates comes exclusively from the number of microstates that correspond to each macrostate, and not from differences in energy between microstates. Although also present in thermal systems, this entropic aspect is especially clear in the simulation of athermal ones: the entire evolution and selection of a given polymorph is not related in any way to differences in internal energy *U* (which can only be 0 or *∞*, for feasible or infeasible configurations, respectively), but solely to the number of feasible configurations. The stable polymorph turns out to be the one with the (overwhelmingly) larger number of microstates.

### 3.2. Chain Statistics and Conformations

**Figure A1:**
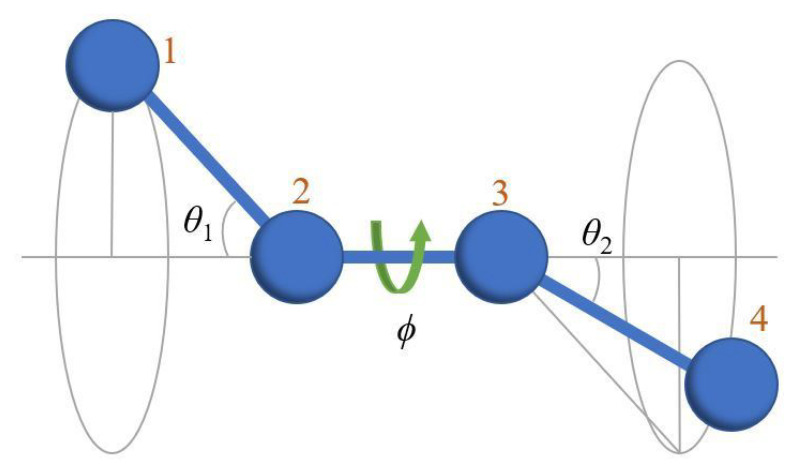
Definition of bending angles $\theta_{1},\theta_{2}$, and torsion angle ϕ. The angle ϕ is measured with respect to the plane defined by bonds 1–2 and 2–3.

An inspection of system frames shows that as the simulation progresses, monomers tend to occupy (on average) the sites of crystalline clusters. This requirement has an impact on the bending and torsional angles along the backbone (see a sketch of Figure A1 for the definition of bending and torsion angles), for chains with unrestricted torsional and bending angles cannot efficiently occupy the sites of a crystal.

**Figure 7:**
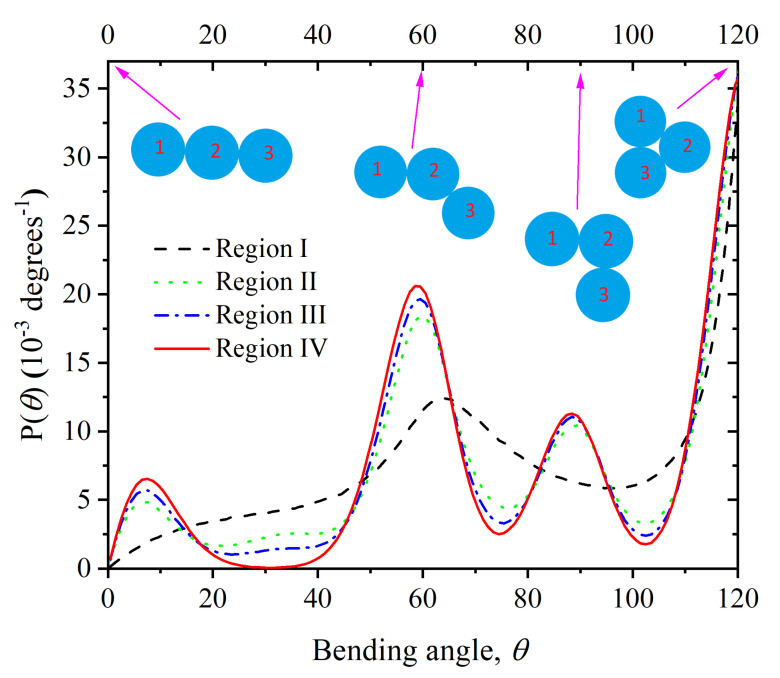
Probability distribution function of bending angles averaged over Regions I through IV. Values of $\theta >120^{\circ }$ are forbidden, due to an overlap of monomers 1–3. Three-site arrangements that correspond to specific bending angles are sketched as indicated by the arrows. The following color and style format is used throughout the manuscript: Region I (dashed black); Region II (dotted green); Region III (dashed-dotted blue); Region IV (solid red).

The distribution of bending angles in the crystal displays a good deal more structure than in the initial, amorphous state. Individual peaks in the distribution in Region IV can easily be assigned to angles formed by two successive bonds joining three sites in the ideal FCC crystal. In particular, the peaks at $60^{\circ }$ and $120^{\circ }$ are due to parts of the chains laying on sites of the 2D compact hexagonal layers from which the FCC crystal is built up. The broad peaks in Figure 7 that correspond to specially simple arrangements of three consecutive monomers which occur with high probability, are shown as sketches in the same figure. The arrows in Figure 7 mark the values of the bending angles for these simple arrangements. The bending angle values $\approx 30^{\circ },75^{\circ },$ and $105^{\circ }$ are strongly suppressed in the crystal because they do not match the geometry of three consecutive sites along a chain on the FCC crystal. The angles of θ=33.5 and $70.5^{\circ }$ have been found to play an important role in the crystallization of bend-core trimers interacting with the Lennard-Jones potential [115]. These angles are also unique for the HCP crystal, compared to the angles of θ=0,60,90, and $120^{\circ }$, which are common to both the HCP and FCC crystals. In our simulations at high densities, these bending conformations are improbable also in the initial amorphous phase, as they correspond to obtuse angles that do not minimize the local volume for triplets of monomers along the chain backbone. As crystallization proceeds, their population diminishes as a combined effect of the hard sphere model for the monomer interactions and the freely jointed model for chain connectivity.

**Figure 8:**
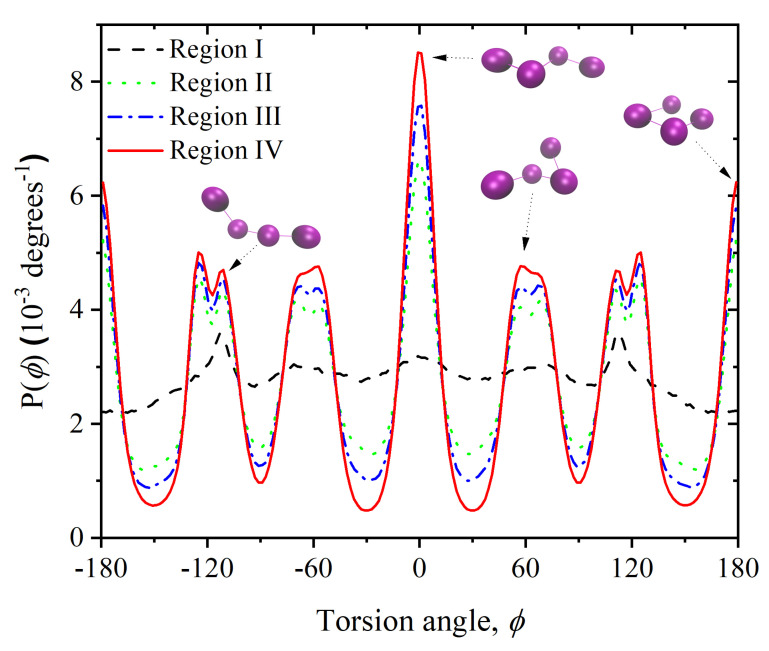
Probability distribution of torsion angles averaged over Regions I through IV. Value of 0${}^{\circ }$ corresponds to the *trans* conformation. Also shown are four-site arrangements that correspond to specific torsion angles, as indicated by the arrows.

As Figure 8 shows, the distribution of torsional angles (ϕ defined by monomers 1-2-3-4 in Figure A1) in the crystal also strongly deviates from the flat distribution of individual, isolated chains, and also from the distribution of the initial, amorphous configuration. Wide, bimodal peaks result from the overlap of closely spaced, favored torsion angles, like $54^{\circ }$ and $60^{\circ }$. The peaks in the torsion angle distribution also correspond to specific conformations of four consecutive monomers (three bonds) whose positions correspond to neighboring sites in an FCC crystal. The torsion angles compatible with an ideal FCC crystal correspond to the values of ϕ=0, 54.7, 70.5, 90, 109.5, 125.3, and $180{}^{\circ }$. Some of these four-monomer sequences on the sites of a perfect FCC crystal are sketched along the torsion angle distribution in Figure 8. The virtually perfect symmetry about $\phi =0{}^{\circ }$ of the distribution in Figure 8 is further proof of the robust configurational sampling due to the efficiency of the MC protocol and the great length of the simulation. It is further evident that for both the bending and torsion angle distributions, the minima and maxima become progressively more pronounced as crystal perfection advances, which is consistent with the observation that monomers tend to occupy, within fluctuations, the sites of an FCC crystal.

**Figure A2:**
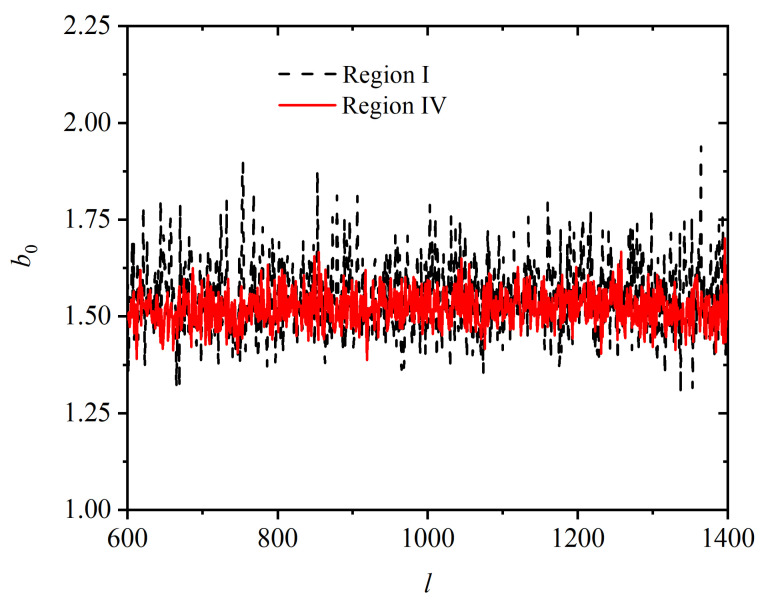
Plot of Kuhn length $b_{0}$ as a function of chain length averaged over Regions I and IV. Within statistical uncertainty, the Kuhn length $b_{0}=1.52\pm 0.05$ is independent of the chain length $l\in [l_{min},l_{max}]$, and is the same for both regions.

A key question that arises is whether chains with torsional and bending angles distributions, that so markedly deviate from those in the amorphous state, also have unusual overall conformational properties. Ideal chain behavior in the crystal may at first sight seem counterintuitive and incompatible with the fact that monomers occupy the sites of a crystal and with the far from uniform distributions of bending and torsion angles. On the one hand, the great length of the chains renders local conformational details imposed by the geometry of the FCC crystal irrelevant. Still, there is another reason for the chains to display ideal behavior. As will be qualitatively discussed in the next Section (and quantitatively in a companion paper [94]), the stable macrostate for the polymer system is a crystal because it maximizes the sum of monomer translational entropy, because they occupy the sites of a crystal, and chain conformational entropy. This is because chains adopt ideal conformations compatible with monomers occupying the sites of the crystal. Thus, the crystal results from a combination of maximal positional freedom for the individual monomers about the sites of an ideal crystal, and maximal conformational variability for the entire chains. This result is independent of chain length, even for chains of moderate length (l≈10) [71]. The chains in the present study are far longer. Confirmation that all chains in the system are deep in the polymeric regime is also provided by a plot of Kuhn length $b_{0}$ in Figure A2.

**Figure 9:**
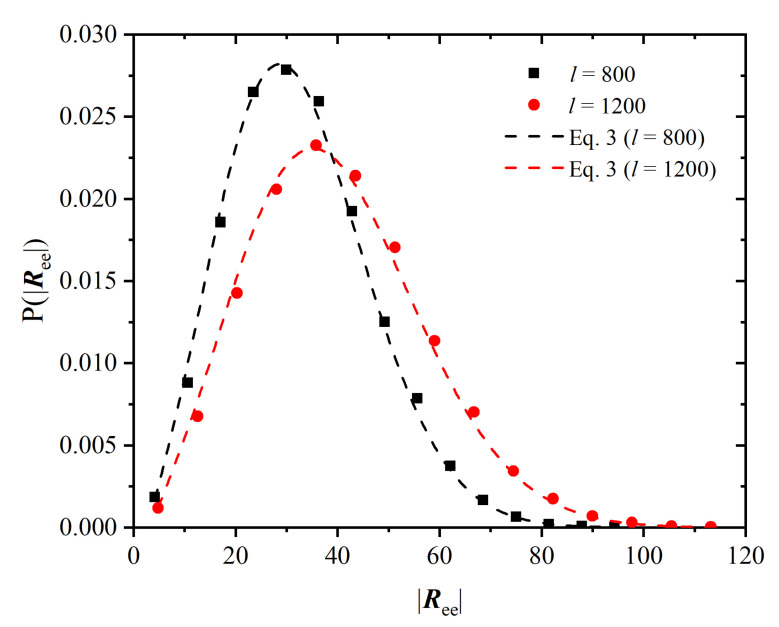
Probability distribution of the modulus of the end-to-end vector, $|R_{ee}|$ averaged over the production phase, Region IV, for chains of lengths l=800±50 and l=1200±50 (small intervals of *l* instead of single values have been used to obtain better statistics), together with the expected distributions according to Equation (2), setting Kuhn length $b_{0}=1.52$, according to Figure A2.

Figure 9 shows the probability distribution function of the end-to-end vector modulus $|R_{ee}|$ for chains of lengths l=800±50 and l=1200±50 (scatter symbols), together with the expected distributions (dashed lines) for the same chain lengths, given by $4\pi |R_{ee}{|}^{2}f\left(R_{ee}\right)$ with:

(2) $$\begin{array}{c} f\left(R_{ee}\right)={\left(\frac{3}{2\pi lb_{0}}\right)}^{\frac{3}{2}}\exp\left(-\frac{3|R_{ee}{|}^{2}}{2lb_{0}}\right) \end{array}$$

being the distribution of the end-to-end vector, and σ=1 has already been taken into account in (2). In addition, the dashed lines are curves of the same functional form (2), fitted to the distributions obtained from the simulation. Gaussian behavior is clearly observed for both chain lengths in the FCC crystal in accordance with the ideal random-walk behavior.

### 3.3. Entropic Origins of Crystallization

The space available to monomers, its homogeneity and its isotropy, can conveniently be analyzed by means of the Dirichlet tesselation of space in Voronoi polyhedra, based on the coordinates of the monomers, as described in the context of the CCE-norm.

**Figure 10:**
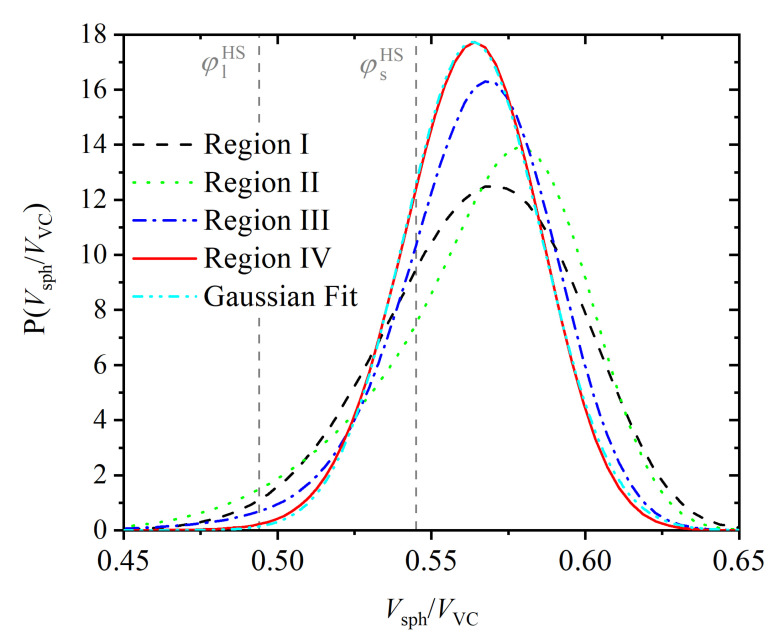
Local volume fraction distribution for the four different regions. The local volume fraction for a given monomer is defined as the ratio of the volume of a monomer ($V_{sph}=\frac{\pi }{6}$) to the volume of its Voronoi cell $V_{VC}$. The average of all distributions is identical and coincides with the overall volume fraction φ=0.56. The equilibrium distribution (Region IV) follows the Gaussian behavior to excellent accuracy, as can be seen by the corresponding fit, with 0.563 and 0.0227 being the mean and standard deviation, respectively. Vertical dashed lines mark the volume fractions of the liquid and solid phases of the monomeric HS fluid at equilibrium, $\varphi_{l}^{HS}=0.494$ and $\varphi_{s}^{HS}=0.545$, respectively.

The increase in the regularity of the volume available to the monomers as the crystal is formed can be observed in Figure 10. This figure shows the probability distribution of the local density (measured as the ratio of the volume of a monomer ($V_{sph}=\frac{\pi }{6}$) to the volume of its Voronoi cell ($V_{VC}$) for Regions I through IV. Since the $\left[VTN_{sites}\mu^{*}\right]$ ensemble is isochoric, the curves in Figure 10 have the same mean value, but their shapes and variances do change in the course of the simulation. The wider initial distribution reflects the greater abundance of monomers with either low or high local volume fraction (i.e., high or low volume of their Voronoi cell), while the narrower distribution in Region IV highlights the greater spatial homogeneity (in the sense of the distribution having smaller variance) of the volume of the Voronoi cells in the FCC state. The distribution in Region IV (crystal) follows a Gaussian distribution almost perfectly (dashed line in Figure 10), which is consistent with the fluctuations of the monomers about the sites of an FCC crystal [116,117]. For reference purposes, the local volume fractions of the liquid and solid phases of the *monomeric* HS fluid at equilibrium are 0.667 and 0.736 times the maximum close packed density (0.7405), i.e., $\varphi_{l}^{HS}=0.494\;\left({\varphi_{l}^{*}}^{HS}=0.667\right)$ and $\varphi_{s}^{HS}=0.545\;\left({\varphi_{s}^{*}}^{HS}=0.736\right)$, respectively [42,118] (marked with vertical lines in Figure 10).

A clearer insight into the mechanism of the phase transition is given by the evolution of the volume *accessible* ($V_{ac}$) to a monomer within its own Voronoi cell. A simple yet convenient definition of accessible volume is that of the region of the Voronoi cell within which the center of the spherical monomer can be placed while keeping its distance to the nearest face of the Voronoi polyhedron <0.5. The accessible volume in each Voronoi cell is computed here via straightforward MC integration. Although much more refined methods exist, like the analytical treatment in [119], the present approach is sufficiently precise for the calculations.

**Figure 11:**
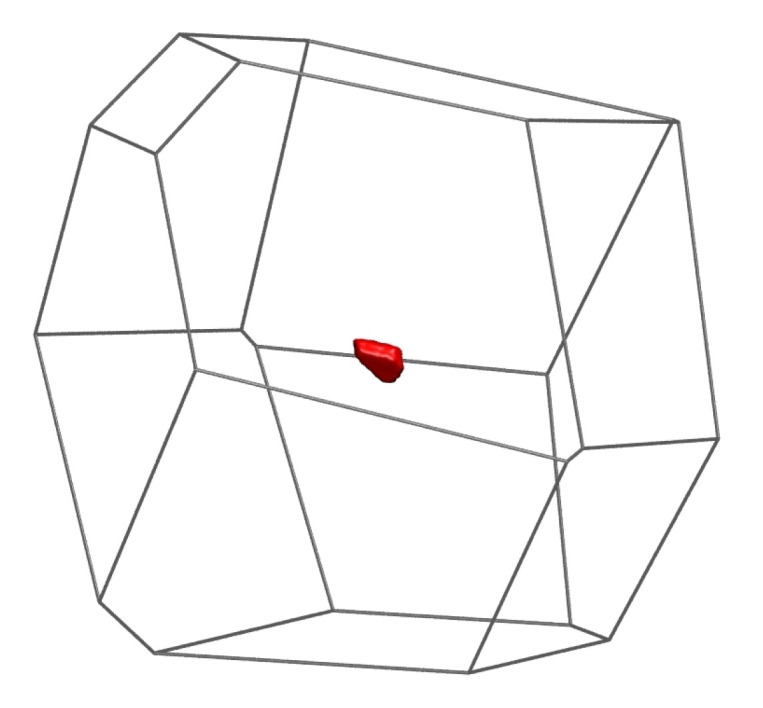
Volume accessible to a monomer with $\varepsilon^{FCC}=0.18$ within its Voronoi cell. For this monomer: $V_{ac}/V_{VC}=0.00024$. Accessible volume is colored in red and the Voronoi polyhedron is shown in wireframe representation.

**Figure 12:**
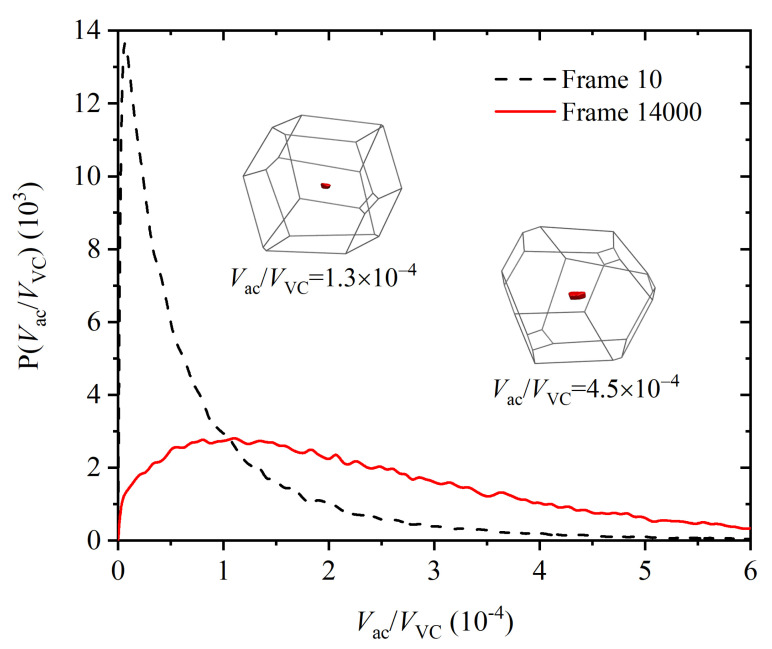
Relative local accessible volume distribution in the amorphous state (black dashed line) and in the crystalline state (red solid line). Relative accessible volume is the ratio of the local accessible volume of a monomer to the volume of the Voronoi cell of the same monomer. The distributions of the absolute accessible volume follow the same trend. Two snapshots of different accessible volumes from sites of the last recorded configuration (frame 1400) are shown as examples.

The shape of the accessible volume is a smaller version of the Voronoi polyhedron, but with slightly rounded vertices, and whose faces are parallel to the faces of the Voronoi polyhedron. The logarithm of its size gives, to first order [120,121,122], a quantitative measure of the translational entropy of the monomer, under the neglection of multibody correlation and conformational entropy; that is, ignoring the fact that it belongs to a polymeric chain. Figure 11 shows a typical Voronoi cell as a wireframe representation, and the accessible volume as the red solid shape within the Voronoi cell. Unlike the total system volume *V*, the total accessible volume $V_{ac}$ is not conserved and is very sensitively related to changes in translational entropy. Figure 12 shows the distribution of the accessible volume for all $N_{sites}=54,000$ monomers, in the initial amorphous state at the beginning of the MC calculation, early in Region I and in the last frame of Region IV, i.e., when the FCC polymorph has fully developed. The distribution of accessible volume becomes clearly narrower, and its mean value shifts to the right. The mean relative accessible volume $\langle V_{ac}/V_{VC}\rangle$ almost triples from $9.0\times 10^{-5}$ to $2.5\times 10^{-4}$ as the system crystallizes. The whole distribution shows this increase vividly (Figure 12), and is direct evidence that, by adopting a spatially more homogeneous and geometrically more isotropic arrangement in the crystal, monomers increase their translational freedom, and hence, the system entropy. This is the main driving force for the crystallization of the chains, just as in a system of monomeric hard spheres.

**Figure 13:**
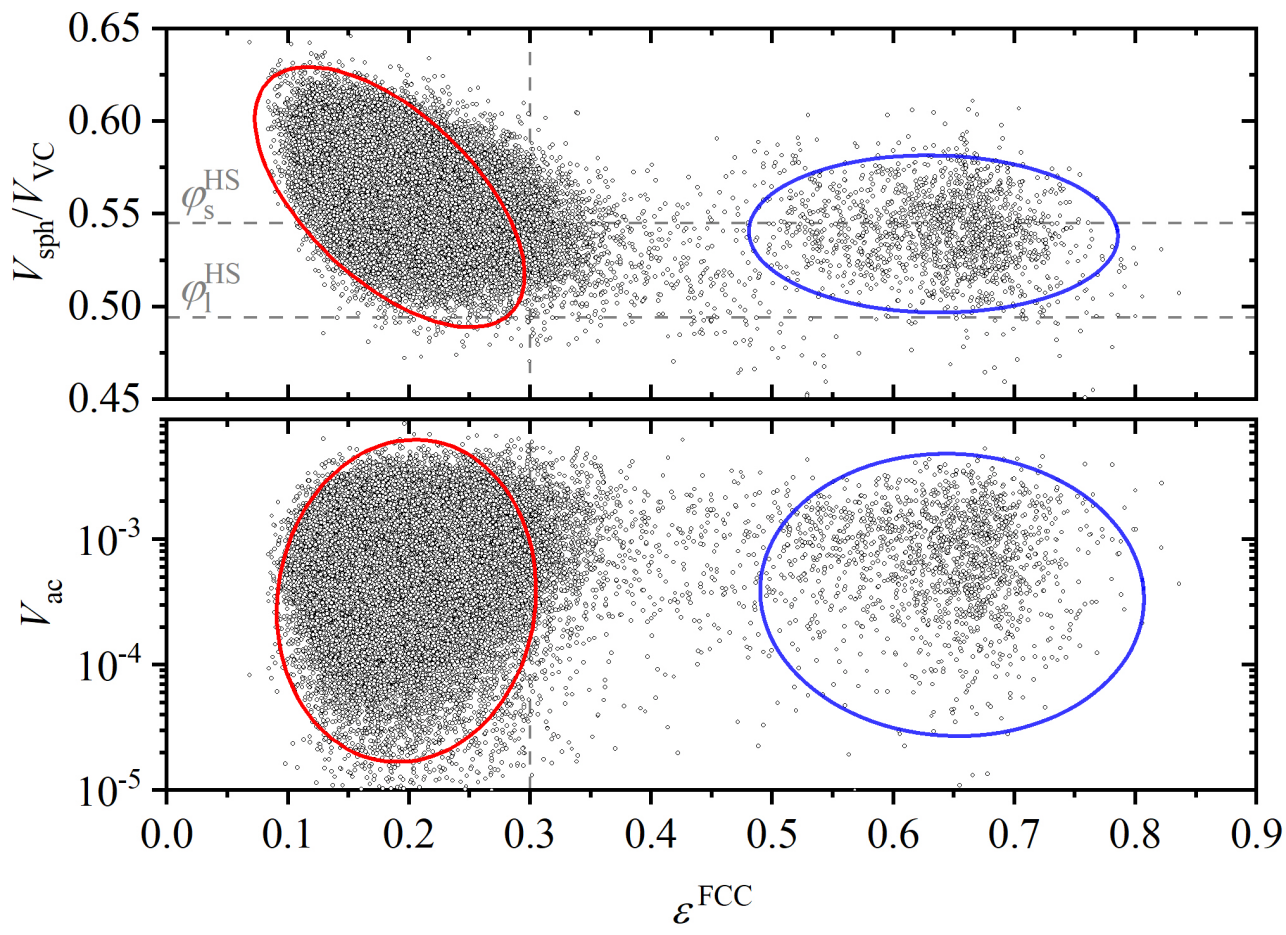
Parity plot of local volume fraction (upper panel) and accessible volume (bottom panel) at frame 14,000 in Region IV vs. the corresponding FCC-CCE norm, $\varepsilon^{FCC}$. Red ellipse denotes FCC sites. Blue ellipse corresponds to non-FCC sites. Horizontal dashed lines in the upper panel mark the volume fractions of the liquid and solid phases of the *monomeric* HS fluid at equilibrium, i.e., $\varphi_{l}^{HS}=0.494$ and $\varphi_{s}^{HS}=0.545$, respectively. The vertical dashed line denotes the CCE-norm threshold $\varepsilon^{thres}=0.3$.

The entropic driving force for crystallization can also be gauged by the correlation between local density and crystal quality, as measured using the CCE norm. Low values of this norm $\varepsilon^{FCC}$, i.e., high local similarity to the perfect FCC crystal, are correlated with high local density, as Figure 13 demonstrates. In Figure 13, data points with $\varepsilon^{FCC}$ values below 0.30 have quite a perfect FCC crystalline environment. This numerical value separates the monomers into two non-overlapping clusters, one for non-FCC sites, in which no correlation between density and crystal quality is apparent, and a much more numerous second one, in which the correlation between density and crystal quality is very marked. As expected, monomers with an almost perfect FCC environment have significantly higher local density. The bottom panel in Figure 13 documents the wide range of accessible volumes for FCC sites: the accessible volume $V_{ac}$ for sites of very similar and quite perfect FCC character (with an average $\varepsilon^{FCC}\approx 0.2$) spans a range of two and a half orders of magnitude. This distribution is of course not static: the accessible volume of a given monomer fluctuates along the simulation within the approximate range $\left[2\times 10^{-5},6\times 10^{-3}\right]$ while keeping an almost constant value of $\varepsilon^{FCC}$, i.e., a quite perfect FCC character.

This is in complete agreement with the entropic origin of the transition: even though local density (inversely related to Voronoi cell volume) increases upon crystallization, monomer translational entropy increases because the accessible volume increases on average. The broad distribution for Region I in Figure 10 (black line) implies that an appreciable number of sites have very small Voronoi cells and hence very little accessible volume (black line in Figure 12).

**Figure 14:**
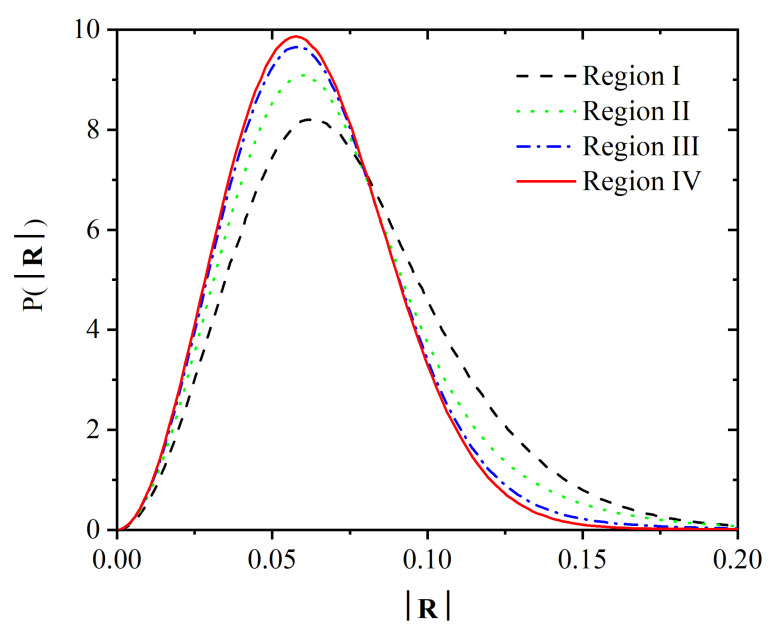
Distribution of the modulus of the vector joining the position of a given monomer and the centroid of its Voronoi cell, |R|, for the four different regions.

During crystallization, Voronoi polyhedra not only become more homogenous in size, (as evidenced by the narrower distribution in Figure 10 and the shifted distribution in Figure 12 for Region IV), but also more isotropic. We have monitored the change in Voronoi cell isotropy in two complementary ways. A first straightforward measure is given by the modulus |R| of the vector joining the position of a given monomer and the centroid of its Voronoi cell. As crystallization takes place, the monomer and the centroid of its Voronoi cell tend to be closer together on average (shift to the left in the mean of the distribution of |R| in Figure 14), but also the distribution of |R| becomes narrower.

A resolved view in the change of the Voronoi cells is further provided by the evolution of the descriptors quantifying the shape of the Voronoi polyhedra, as derived from the second-order gyration tensor (see definition and corresponding results in Appendix A.3). In a rather literal sense, the increase in the accessible volume within Voronoi polyhedra, accompanied by greater uniformity and isotropy, is the force driving the transition from the amorphous to the perfect FCC crystal state, in spite of the opposition due to a loss of chain conformational entropy.

## 4. Conclusions

We have investigated the stability of crystal polymorphs for freely jointed chains of hard spheres deep in the polymeric regime through unprecedentedly long simulations, based on Monte Carlo algorithms. These simulations shed light on the competition between crystal polymorphs and on crystal perfection. Structural characterization of the local environment around each site allowed for a precise identification of crystal morphologies, which range from the early, defect-ridden, cyclic twin structures of mixed HCP/FCC type to the final, highly perfect, stable FCC crystal. We find the stable polymorph macrostate to be highly degenerate: all realizations (microstates) of the stable FCC macrostate consist of chains whose monomers tend to occupy, within spatial fluctuations, the sites of the ideal FCC crystal, while maintaining the constraints of chain connectivity and bond length. Polymer chains nevertheless display ideal behaviors, as their great lengths render local conformational details imposed by the geometry of the FCC crystal irrelevant.

The present simulations allow us to identify the entropic origin of the phase transition: the loss of chain conformational entropy is more than compensated for by the increase in translational entropy as the accessible volume within the Voronoi polyhedra increases. We have also carried out an analysis of accessible volume that provides further insights into the changes in homogeneity and isotropy of Voronoi cells responsible for the increase in monomer translational entropy. This increase in translational entropy is still large enough to drive the phase transition, in spite of chain conformational loss.

In addition, the present MC results also provide a basis for the calculation [94] of the entropic advantage of the FCC with respect to the HCP polymorph. The proposed methodology is currently being extended to tackle semi-flexible athermal polymers in the bulk and under confinement, as well as in composites with colloidal nanoparticles in the form of spheres and cylinders of varied size.

## Figures and Tables

**Figure A3:**
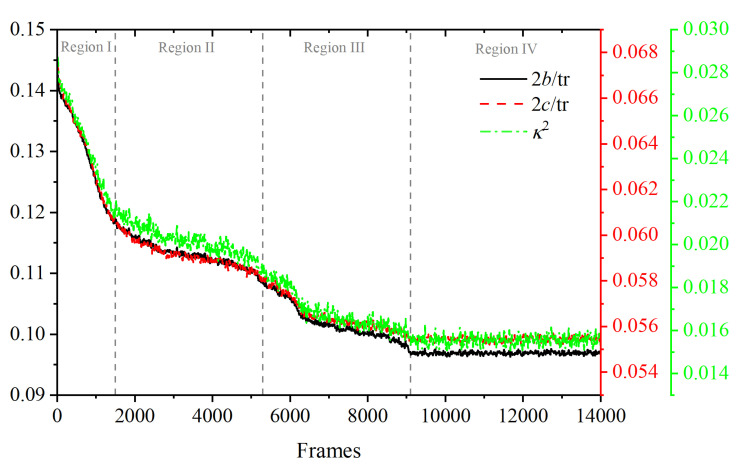
Shape descriptors asphericity *b*, acylindricity *c*, and relative shape anisotropy $\kappa^{2}$. The first two are made dimensionless by dividing by half the trace of G.

**Figure A4:**
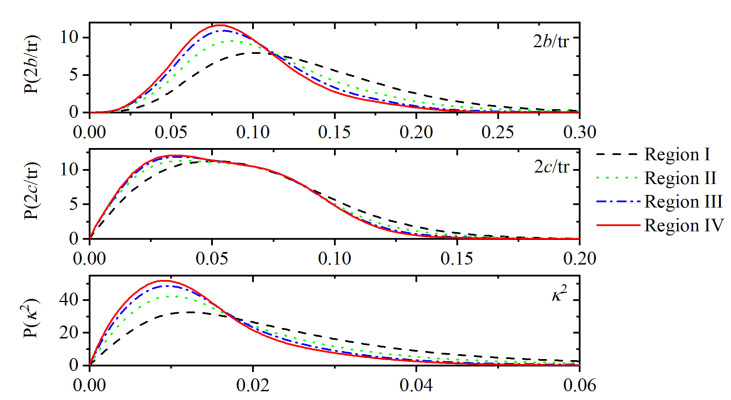
Probability distributions of shape descriptors *b*, *c*, and $\kappa^{2}$ for Regions I and IV. The first two are made dimensionless by dividing by half the trace of G.

## Data Availability

The presented simulation data are fully available upon request.

## Supplementary Materials

The following supporting information can be downloaded at: https://www.mdpi.com/article/10.3390/polym14204435/s1. Video showcasing the evolution of fivefold sites in Regions I and II (first 5000 frames of the MC simulation).

## Abbreviations

The following abbreviations are used in this manuscript:

AMO	Amorphous
BCC	Body-Centered Cubic
CCAMs	Chain-Connectivity-Altering Moves
CCE	Characteristic Crystallographic Element (norm)
FCC	Face Centered Cubic
FIV	Fivefold
HCP	Hexagonal Close Packed
HS	Hard Sphere
KMC	Kinetic Monte Carlo
MC	Monte Carlo
MD	Molecular Dynamics
PBC	Periodic Boundary Condition
rHCP	Random Hexagonal Close Packed
VC	Voronoi Cell
List of variables
*b*	asphericity
$b_{len}$	bond length
$b_{0}$	Kuhn length
*c*	acylindricity
dl	mumerical tolerance in bond length
G	second-order gyration tensor
*I*	position index
*k*	Boltzmann constant
$l,l_{ave},l_{min},l_{max}$	chain length, average, minimum-, maximum chain length
$n_{dis}$	number of trials in configurational bias schemes
$n_{FIV}$	number of fivefold sites
*N*	number of polymer chains
$N_{frames}$	number of frames
$N_{sites}$	total number of monomeric sites
$N_{V}$	number of vertices of the Voronoi polyhedron
$r_{ij}$	distance between monomers *i* and *j*
$r_{m}$	position vector of a monomer.
R	displacement from Voronoi Cell centroid vector
$R_{g}$	radius of gyration
$R_{ee}$	end-to-end distance
*s*	entropy
$S^{X}$	order parameter for crystal type *X*
*T*	temperature
$u_{HS}$	hard sphere interaction energy
*U*	internal energy
$V,V_{ac},V_{sph},V_{VC}$	volume, accessible volume, monomer volume, Voronoi cell volume
$V_{a}$	accessible volume
Greek symbols
$\Delta s_{m}^{conf}$	conformational entropy difference per monomer
$\Delta s_{m}^{trans}$	translational entropy difference per monomer
$\varepsilon^{X}$	CCE-norm with respect to crystal type *X*
ϕ	torsion angle
$\kappa^{2}$	relative shape anisotropy
$\lambda_{1}^{2},\lambda_{2}^{2},\lambda_{3}^{2}$	eigenvalues of G
φ	volume fraction
$\varphi^{*}$	volume fraction relative to maximum HS packing density (0.7405)
σ	monomer diameter
μ	chemical potential
ν	Flory exponent
$\tau_{c}$	total crystallinity
θ	bending angle

## Appendix A

### Appendix A.1. Definition of Bending and Torsion Angles

Figure A1 schematically shows the definition of bending and torsion angles, as adopted in the present work.

### Appendix A.2. Kuhn Length and Ideal Chain Statistics

A useful measure of chain coiling is given by Kuhn’s bond length or by the equivalent freely jointed, phantom chain (i.e., without any kind of excluded volume interactions) [123]. This equivalent phantom chain has the same contour length and the same squared end-to-end distance as the original chain (with excluded volume through Equation (1)). In order to fulfill these two conditions, the equivalent chain has two degrees of freedom: the number of equivalent segments $l^{\prime }$ and its bond length, also known as the Kuhn length, $b_{0}$.

From these two conditions:

$$\begin{array}{c} \left.\begin{array}{c} l^{\prime }b_{0}=l\langle b_{len}\rangle \\ l^{\prime }b_{0}^{2}=\langle |R_{ee}{|}^{2}\rangle \end{array}\right\}\Rightarrow b_{0}=\frac{\langle |R_{ee}{|}^{2}\rangle }{l\langle b_{len}\rangle } \end{array}$$

where $\langle |R_{ee}{|}^{2}\rangle$ is obtained for all chain lengths.

Figure A2 shows a plot of Kuhn length $b_{0}$ as a function of chain length in Regions I and IV. The independence of Kuhn length $b_{0}$ on chain length indicates that even the shortest ($l=l_{min}=600$) chains in the system behave as ideal and fully flexible chains, and that all chains in the system can be characterized by the same Kuhn length $b_{0}=1.52\pm 0.05$ (taking the bond length as a unit).

The very small value of the Kuhn length (≈1.5 times the bond length) indicates an extremely high degree of chain coiling. Interestingly, no particular deviation in $b_{0}$ is detected between the initial, amorphous state (early part of Region I) and the perfected FCC chain crystal (Region IV), even if the bonded geometry undergoes significant changes during the transition, as demonstrated in Figure 7 and Figure 8. The only appreciable difference is the slightly larger variance in the distribution of $b_{0}$ (larger fluctuations of the dashed curve in Figure A2) for different chain lengths, as expected from the greater spatial heterogeneity of the system in the amorphous state.

### Appendix A.3. Shape Measures of the Voronoi Polyhedra

For a given Voronoi polyhedron, we define G as the averaged dyadic of the position vectors of its vertices with respect to the centroid:

(A1) $$\begin{array}{c} G=\frac{1}{N_{V}}RR \end{array}$$

i.e., the number-averaged analog of the moment of inertia tensor of a set of points of unit mass placed at the vertices of the Voronoi polyhedron.

Several scalar shape measures can be extracted from G, such as the asphericity *b*, acylindricity *c*, and the relative shape anisotropy $\kappa^{2}$ coefficient, which are defined, respectively by:

(A2) $$\begin{array}{cc} \; & b=\lambda_{1}^{2}-\frac{1}{2}\left(\lambda_{2}^{2}+\lambda_{3}^{2}\right)\;c=\lambda_{2}^{2}-\lambda_{3}^{2}\\ \; & \kappa^{2}=\frac{3}{2}\frac{\lambda_{1}^{4}+\lambda_{2}^{4}+\lambda_{3}^{4}}{{\left(\lambda_{1}^{2}+\lambda_{2}^{2}+\lambda_{3}^{2}\right)}^{2}}-\frac{1}{2} \end{array}$$

where $\lambda_{1}^{2},\lambda_{2}^{2}$, and $\lambda_{3}^{2}$ are the three eigenvalues of the positive definite G, ordered such that $\lambda_{1}^{2}>\lambda_{2}^{2}>\lambda_{3}^{2}$.

Notice that while $\kappa^{2}$ is dimensionless, *b* and *c* have dimensions of length squared; they are usually made dimensionless through division by $\frac{1}{2}G$. The lower the values of *b*, *c*, and $\kappa^{2}$, the more isotropic the shape of the Voronoi polyhedron. An axisymmetric point distribution has c=0, while a spherically symmetric one has $b=c=\kappa^{2}=0$. The Voronoi polyhedron for the FCC crystal, the rhombic dodecahedron, and for the HCP crystal, the trapezo-rhombic dodecahedron, both also have $b=c=\kappa^{2}=0$.

In Figure A3, we show the evolution of the (dimensionless) *b*, *c*, and $\kappa^{2}$ along the entire MC simulation. All shape descriptors (A2) in Figure A3 decrease monotonically from the initial values as the simulation evolves (the relative drops are b≈33%, c≈17%, and $\kappa^{2}\approx 43\%$), and remain constant (within fluctuations) in Region IV of the stable polymorph. It is remarkable how faithfully all shape descriptors track the evolution of the systems through the four regions and the final onset of crystallinity. As with accessible volume, the distributions of the shape descriptors (Figure A4) also become narrower, indicating a greater homogeneity of Voronoi cells.
